# Ecological stoichiometry and homeostasis characteristics of plant-litter-soil system with vegetation restoration of the karst desertification control

**DOI:** 10.3389/fpls.2023.1224691

**Published:** 2023-10-06

**Authors:** Tinghui Hu, Kangning Xiong, Yanghua Yu, Jun Wang, Yawei Wu

**Affiliations:** ^1^ School of Karst Science, Guizhou Engineering Laboratory for Karst Desertification Control and Eco-industry, Guizhou Normal University, Guiyang, Guizhou, China; ^2^ Guizhou Oil Research Institute, Guizhou Academy of Agricultural Sciences, Guiyang, Guizhou, China; ^3^ Institute of Pomology Science, Guizhou Academy of Agricultural Sciences, Guiyang, Guizhou, China

**Keywords:** karst desertification control, loquat, ecological stoichiometry, vegetation restoration, internal homeostasis

## Abstract

It is of great significance to clarify the ecologically chemical stoichiometric characteristics of plant-litter-soil in vegetation restoration process for elucidating the nutrient cycling law and soil nutrient management of karst ecosystem. The carbon (C), nitrogen (N) and phosphorus (P) contents of leaves, litter and soil and their stoichiometry were determined in loquat (*Eribotrya japonica*) plantations in a karst plateau canyon after 3, 6, 10 and 15 years of restoration. The homeostasis characteristics of leaf N, P, and N:P with the change in soil nutrients during restoration were revealed. The results showed that leaf C, N, and P contents initially increased and then decreased with increasing years of restoration at the same sampling time. The contents of nutrients in soil and litter varied with increasing restoration years, with the highest values mostly appearing in May and July. This could be due to greater moisture in May and July, which helps with nutrient absorption and transformation. The leaf N:P ratio of loquat with different restoration years was 35.76-47.39, with an average of 40.06. Therefore, loquat leaves may experience P limitation in the growth process. The relationships between N, P and N:P in leaves and soil indexes could be simulated by a homeostasis model. Except for the weak sensitivity of loquat leaf N in 10 years, the other indexes and treatments had a certain homeostasis. Plants maintain homeostasis by regulating physiological responses *in vivo* in response to soil nutrient changes, indicating that loquat has good adaptability in karst desertification environments, but attention should focus on the management of soil P in the field as part of the vegetation restoration process. Therefore, in future research, we should combine the soil water and fertilizer conditions of different growing seasons in karst rocky desertification areas and provide scientific field management to ensure that the results of rocky desertification management can play a role in rural revitalization.

## 1 Introduction

With the Guizhou Plateau as its centre, the karst region of southern China is not only one of the richest landscape types and most complex and typical karst developments but also the most concentrated and largest karst ecologically vulnerable region in the world (Yuan, 1997; Yang, 1998; Xiong et al., 2016). The distribution of carbonate rocks, thin soils, uneven distribution of water and heat conditions under the influence of the monsoon climate, and excessive human cultivation and grazing have led to fragile ecosystems in this area (White, 1988; Ford and Williams, 2007). Reduced biodiversity, severe soil erosion, and low agricultural productivity limit the rapid economic development in karst mountains (Xiong and Chi, 2015; Green et al., 2019). Therefore, rocky desertification management is a major challenge in the region. Loquat (*Eriobotrya japonica* L.) has become an economic tree species used in rocky desertification control because of its drought tolerance, wide adaptability, and high nutritional and economic value. Scientists have achieved significant results in the treatment of rocky desertification by planting loquat trees in the area (Xiong et al., 2002; Xiong et al., 2016; Liu et al., 2021; Hu et al., 2022b; Xiong et al., 2023). However, the lack of scientific management techniques and research on the nutrient elements in the process of vegetation restoration has led farmers to carry out mostly uninformed management, resulting in some plants being shorter and having lower yields. Therefore, it is vital to investigate the plant-litter-soil ecological stoichiometry properties to elucidate the ecosystem nutrient cycling pattern and soil nutrient management in rocky desertification management.

Ecological stoichiometry has become a scientific theory linking biological systems at different levels and scales, reflecting the dynamic balance between energy and chemical elements (Han et al., 2005; Elser and Hamilton, 2007; Sardans et al., 2021). The main research focuses on how the nutrient (mainly C, N and P) requirements of organisms and the supply of nutrients in the environment affect the growth of organisms (Klausmeier et al., 2004; Sardans et al., 2021; Lv et al., 2023). At present, ecological stoichiometry has made theoretical achievements such as the “limiting element stability hypothesis” and plant N:P nutrient restriction in terms of the identification of limiting nutrient elements (Tessier and Raynal, 2003; Elser and Hamilton, 2007). Researchers have carried out many studies on the contents of chemical elements and their ecological stoichiometric traits in soil, litter and plants in ecosystems. In nonkarst areas, such as vegetation restoration areas on the Loess Plateau region (Bai et al., 2019; Deng et al., 2019a; Zhang et al., 2020) and degraded grasslands on the plateau (Zeng et al., 2016; Sun et al., 2019; Wang et al., 2020; Du and Gao, 2021; Wang et al., 2023), scientists have mainly studied the stoichiometric characteristics of soil and plant C, N and P and their nutrient limitations in the different ecosystems. In karst areas, especially in Southwest China, scientists have mainly studied the ecological stoichiometry characteristics of the chemical elements of soil and plants in karst desertification ecosystems (Wang et al., 2018a; Su et al., 2019; Wang et al., 2022; Tang et al., 2023). The majority of studies have been conducted under the conditions of different land use types or different vegetation types (Wang et al., 2018b; Liu et al., 2020; Hu et al., 2022a). There are great differences in ecosystem nutrient contents in different vegetation restoration modes and stages (Li et al., 2015; Ge et al., 2017; Guan et al., 2022). There is a lack of ecological stoichiometric research in different years of vegetation restoration in karst desertification control. The seasonal characteristics of C, N and P ecological stoichiometric changes in plant-litter-soil systems and their driving mechanisms are still unclear.

According to the changes in environmental nutrient elements, organisms improve their adaptability to the environment by adjusting the content and proportion of elements in the body (Sterner and Elser, 2002; Persson et al., 2010). Many scientists have studied the homeostasis regulation characteristics of different populations, including phytoplankton, bacteria, fungi, algae, grassland, crops, and forest species (Levi and Cowling, 1969; Rhee, 1978; Makino et al., 2003; Persson et al., 2010; Yu et al., 2011; Wang et al., 2023). Stoichiometric homeostasis has been reported to be positively correlated with vegetation function and stability (Yu et al., 2010). The climax community had a higher stoichiometric equilibrium intensity than the subdominant species (Hooper et al., 2005). Therefore, the homeostasis of vegetation is related to adaptability. However, the characteristics of plant homeostasis in the control of karst rocky desertification remain to be studied.

Therefore, in this study, we selected loquat (*Eribotrya japonica*), an important tree species for the control of karst desertification in karst plateau valleys, and we used loquat plantations restored for 3, 6, 10, and 15 years as the research objects. The aims were to (1) analyze the seasonal changes in the C, N, and P contents and ecological stoichiometric characteristics of leaf-litter-soil in loquat forests during different restoration years and (2) identify the characteristics of the internal homeostasis of leaf N, P and N:P changes with soil nutrients at different restoration years. This information can clarify the nutrient cycling and homeostasis characteristics of the vegetation restoration system in the ecologically fragile karst desertification area and provide scientific and technological support for the restoration of vegetation and the management of nutrients in the karst desertification control zone.

## 2 Materials and methods

### 2.1 Study region

**Figure 1:**
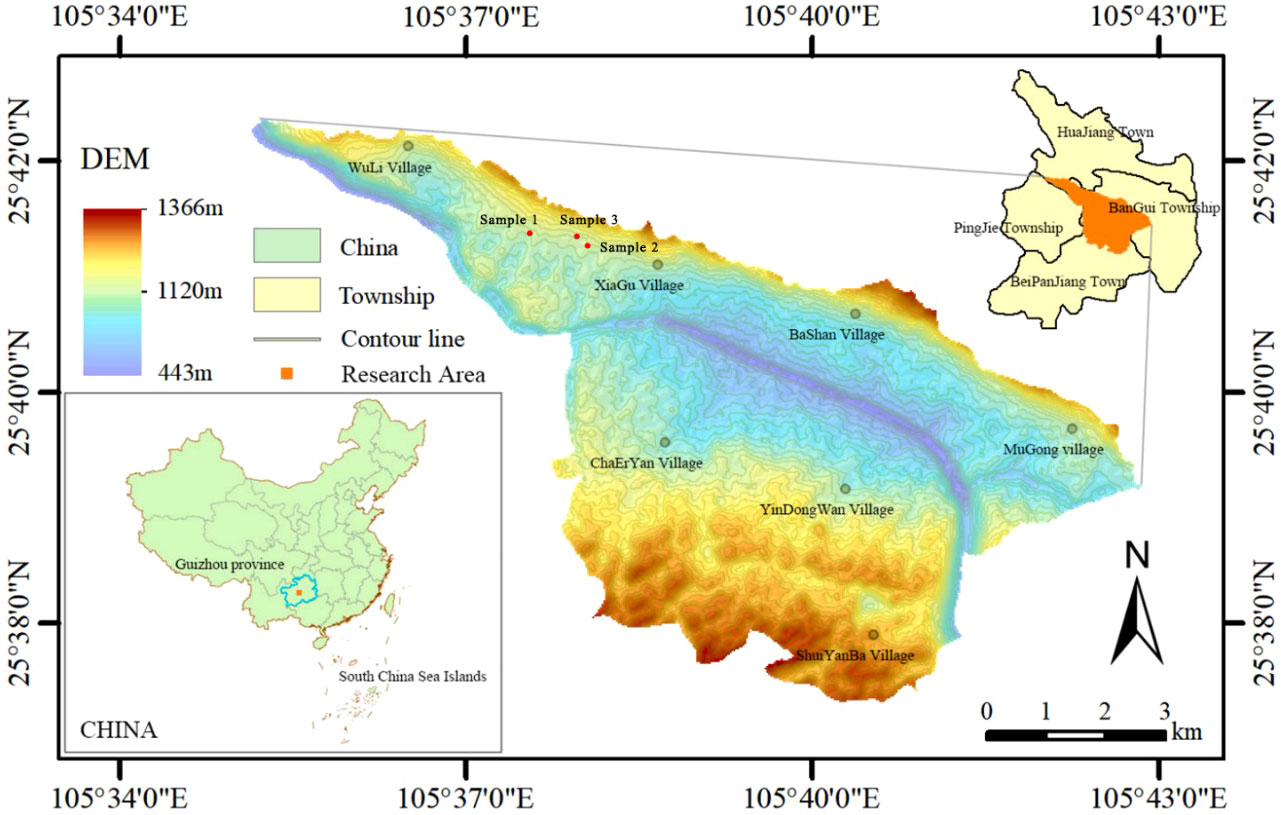
Location of the study area in South China (Wu et al., 2022).

This research was carried out in the Guanling-Zhenfeng Huajiang study area in Guizhou Province, located in the section of Beipanjiang-Huajiang Canyon at the junction of Zhenfeng County, Qianxinan Autonomous Prefecture and Guanling County, Anshun city, Guizhou Province (105°36′30″~105°46′30″ E, 25°39′13″~25°41′00″ N) (**Figure 1**). The comprehensive management demonstration area for desertification of the karst plateau canyon has a total area of 5161.65 hm², and the karst zone is a typical karst plateau canyon area, comprising 87.92% of the total area. The region has a warm subtropical dry valley climate with an uneven distribution of rainfall. Rainfall is concentrated from May to August, and the rainfall in this period accounts for 83% of the total annual rainfall. The average annual temperature remains at 18.4°C, and sufficient light and heat occurs throughout the year. The geomorphological type is dominated by crest depressions, crest valleys, and altitudes of 450-1 450 m. Karst action in the area is intense, resulting in underground fissures, caves, dark rivers, and water caves. The soils are mainly yellow loam and yellow lime soil, with high contents of calcium, magnesium and iron, and the pH value is 7.37. The rocky desertification in the demonstration area has a wide range and a deep degree, and the overall landscape is dominated by medium-intensity rocky desertification, with a rocky desertification area of 13.52 km^2^, of which potential, mild, medium and intense karst desertification account for 24.54%, 40.48%, 17.93% and 17.06%, respectively. After a long period of karst desertification control, the vegetation in the study area has been well restored, mainly by *Zanthoxylum bungeanum*, loquat (*Eribotrya japonica*), *Rosa roxburghii Tratt*, and pitaya (*Hylocereus undatus*).

### 2.2 Experimental design

**Figure 2:**
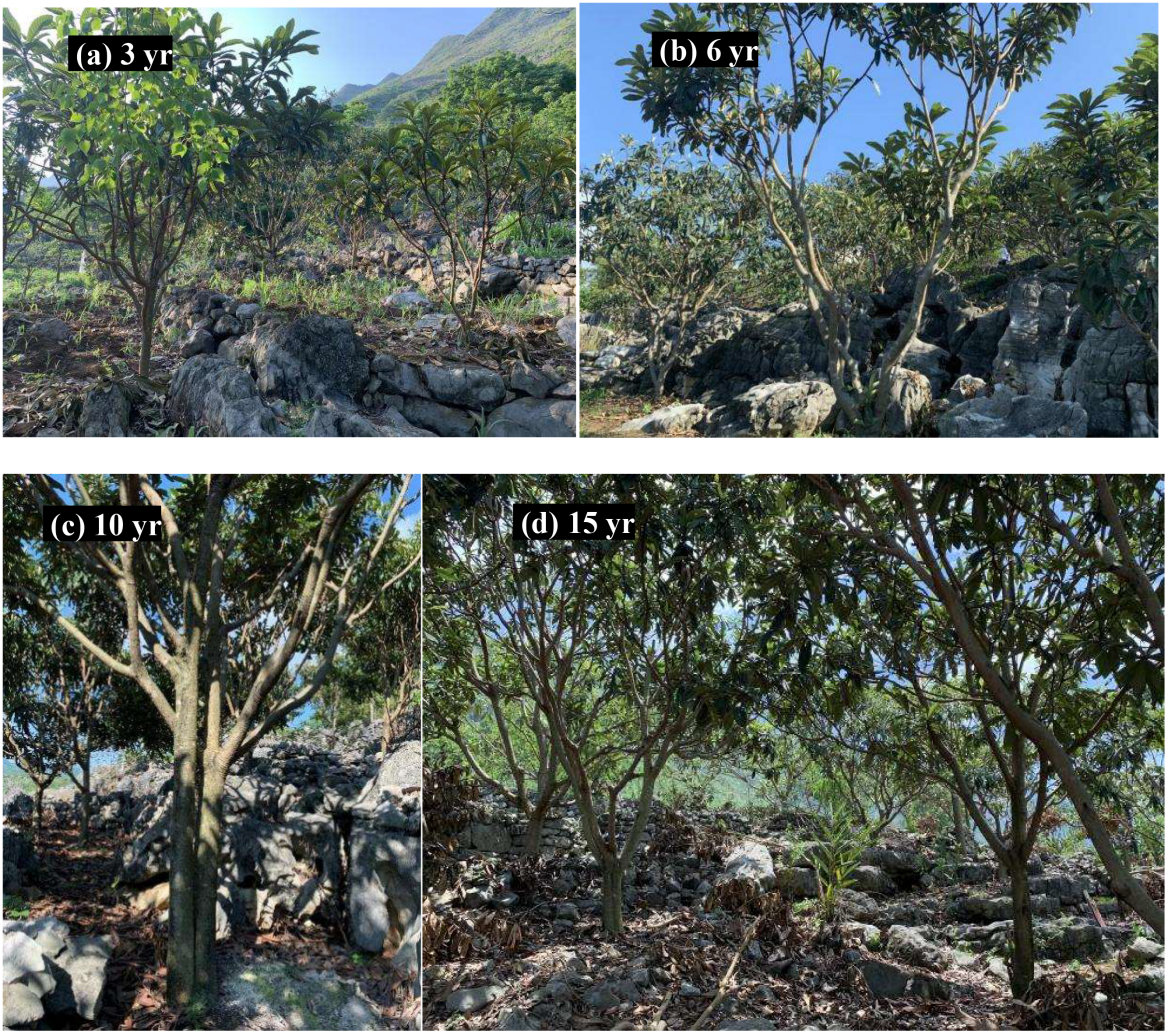
The restorations at 3, 6, 10 and 15 years are shown in photos **(A–D)**, respectively.

Three loquat plots with similar elevations, similar pre-growth environments and essentially the same degree of karst desertification (intense karst desertification) were selected as sample points in February 2021 (**Figure 1**). Four loquats of different recovery years were selected, namely recovered at 3 years (3 yr), 6 years (6 yr), 10 years (10 yr), and 15 years (15 yr) (**Figure 2**). Three standard sample plots of 20 m × 20 m were set for each recovery year, with a total of 36 sample plots, and the distance between sample plots was not less than 10 m. The average tree height, average canopy diameter, and stem diameter at breast height (DBH) of loquats at different recovery ages were shown in **Table 1**.

**Table 1:** The basic growth situation of loquats at four recovery ages.

Recovery year	Mean plant height/m	Mean crown/m	Mean DBH or ground diameter/mm
3 yr	1.70 ± 0.11	1.6×1.6	53.13 ± 2.54
6 yr	3.52 ± 0.13	3.2×3.3	97.82 ± 4.11
10 yr	4.41 ± 0.18	4.3×5.2	136.14 ± 7.75
15 yr	6.22 ± 0.19	6.2×6.5	191.25 ± 9.59

The ground diameter was measured after 3, 6 years of restoration, and the diameter at breast height was measured for the other restoration years.

### 2.3 Sample collection and processing

**Figure 3:**
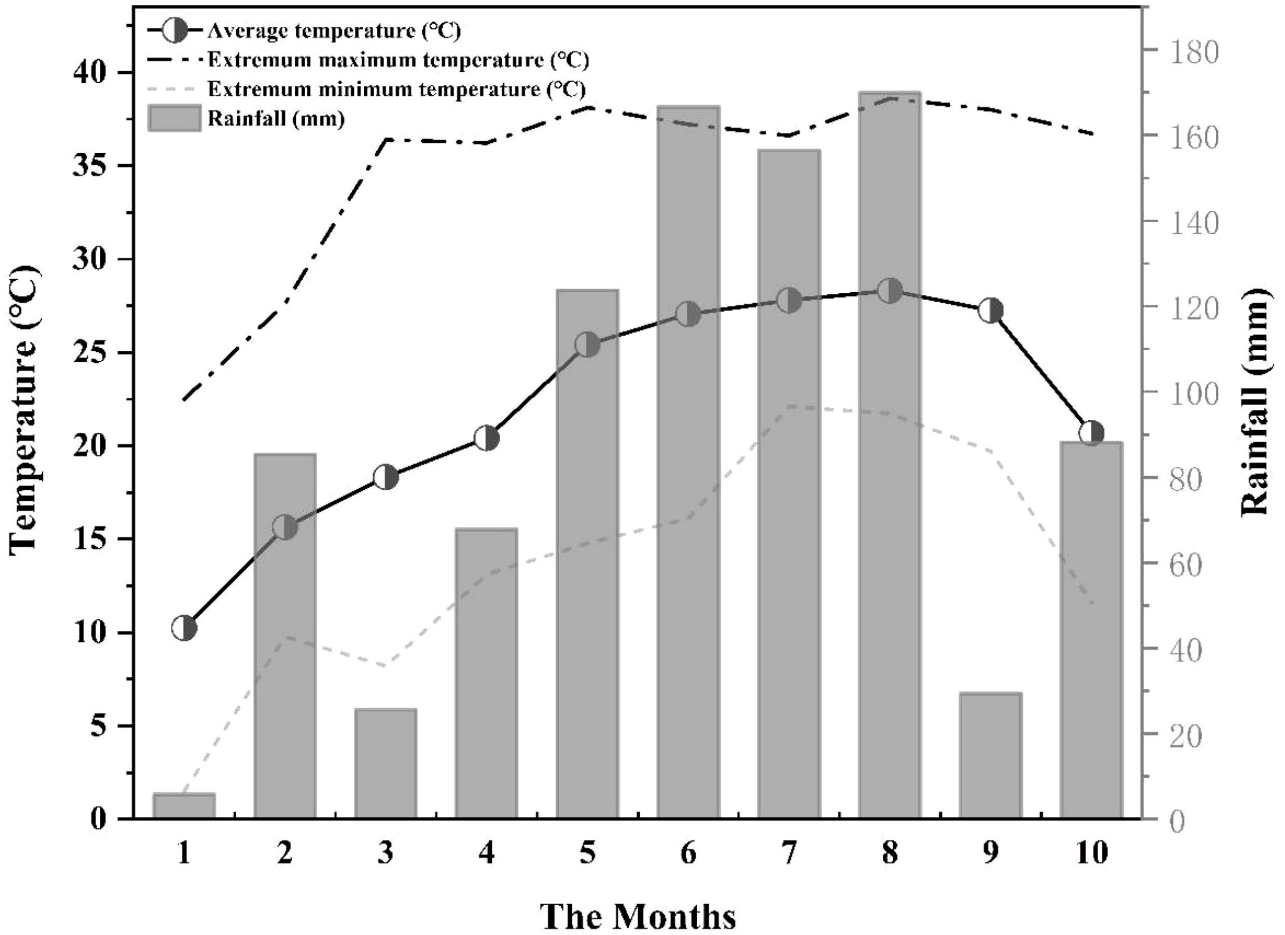
Monthly average rainfall and air temperature during the experiment.

Loquat leaf, soil and litter samples were collected in February (Feb.), May (May), July (Jul.) and October (Oct.) of 2021. The temperature and rainfall during the sampling period from January to October are shown in **Figure 3**. Leaf and litter collection: Five trees with uniform growth were selected from each sample plot. Five leaves were collected from the same parts in the north, south, west and east directions and mixed into nylon mesh bags for preservation. Additionally, sampling squares (1 m × 1 m) were designed from the corresponding five trees in the east, south, west and north directions 30 cm from the trunk to collect litters from the ground and mix them into self-sealing bags. After returning to the laboratory, loquat leaves and litter were washed with clean water and placed in an oven. The oven was set to 105°C, and the leaf and litter samples were dried for 30 minutes and then switched to 70°C until the weight was constant. After crushing with a grinder, they were sieved with a 0.15-mm sieve and kept in a dry and cool place for experimental analysis.

Soil sample collection: At each sample site, soil samples of the 0-20 cm soil layer were collected by a soil auger (50 mm inner diameter) according to the 5-point collection method of the “S” curve (due to the thin soil layer, the actual depth was less than 20 cm). After removing sundries such as stones and roots, the soil samples were evenly mixed. One kilogram of soil sample was packed into a self-sealing bag by the quarter method. After 2 weeks of natural drying in the laboratory, the soil was ground with a ball mill and screened with 0.15-mm and 2-mm screens.

### 2.4 Laboratory analysis

The organic C content of all samples was assayed by the volumetric potassium dichromate method (Bao, 2010). An excessive (K_2_Cr_2_O_7_-H_2_SO_4_) solution was used to oxidize carbon in the samples. After heating, it was titrated with FeSO_4_ (0.2 mol/L) standard solution using phenanthroline as an indicator. The total N of soil (digesting by H_2_SO_4_-HClO_4_) and plant leaves (litter) (digesting by H_2_SO_4_-H_2_O_2_) was measured by the automatic Kjeldahl method (Hanon-K1160, Shandong, China) (Bao, 2010). The total P of soil (digesting by H_2_SO_4_-HClO_4_) and plant leaves (litter) (digesting by H_2_SO_4_) was measured through molybdenum-blue colorimetry (Bao, 2010).

### 2.5 Data analysis

The homeostasis index (*H*) was calculated according to the following calculation method (Persson et al., 2010):


H=lg(x)/(lg(y)−lg(c))

where the dependent variable y is the content of leaf P and N or stoichiometry ratio, the independent variable x is the corresponding soil P and N content or its ratio, and c is the fitting constant. Regression analysis was performed on the P, N, and N:P in loquat leaves at different restoration years. 1/*H* represents the regression slope of lg(*x*) and lg(*y*), and its absolute value ranges from 0 to 1. The larger the value of H is, the more stable and higher the steady state within the organism. If the relationship of regression was not significant (*P* > 0.1), the plant was taken to be ‘rigidly internal-stable’, and the value of 1/*H* was set at zero; if the relationship of regression was significant, i.e., when 1/*H* = 1, the species was considered not homeostatic. Finally, the data sets with 0< 1/*H*< 1 were defined as follows: 1/*H* > 0.75 were sensitive, 0.75 > 1/*H* > 0.5 were weakly sensitive, 0.5 > 1/*H* > 0.25 were weakly homeostatic, and 0.25 > 1/*H* > 0 were homeostatic (Persson et al., 2010).

The soil, litter and leaf P, N and C contents were computed using mass content, and C:P, N:P and C:N were calculated using molar ratios. Experimental results were analysed and processed through SPSS 13.0 and Excel 2016 software; additionally, data sets were assessed for normality before statistical analyses were performed, and log10 transformations were performed to improve normality where necessary. One-way ANOVA (one-way analysis of variance) was adopted to analyze the significance of different sampling times, plants of different restoration years, litter, soil nutrient contents, and ecological stoichiometric ratios. Correlation analysis was performed using Pearson correlation analysis, and the least significant difference (LSD) method was used for multiple comparisons. Plots were made using OriginPro 2021.

## 3 Results

### 3.1 Characteristics of plant, litter, soil C, N and P contents characteristics of plant, litter, soil C, N and P contents

**Figure 4:**
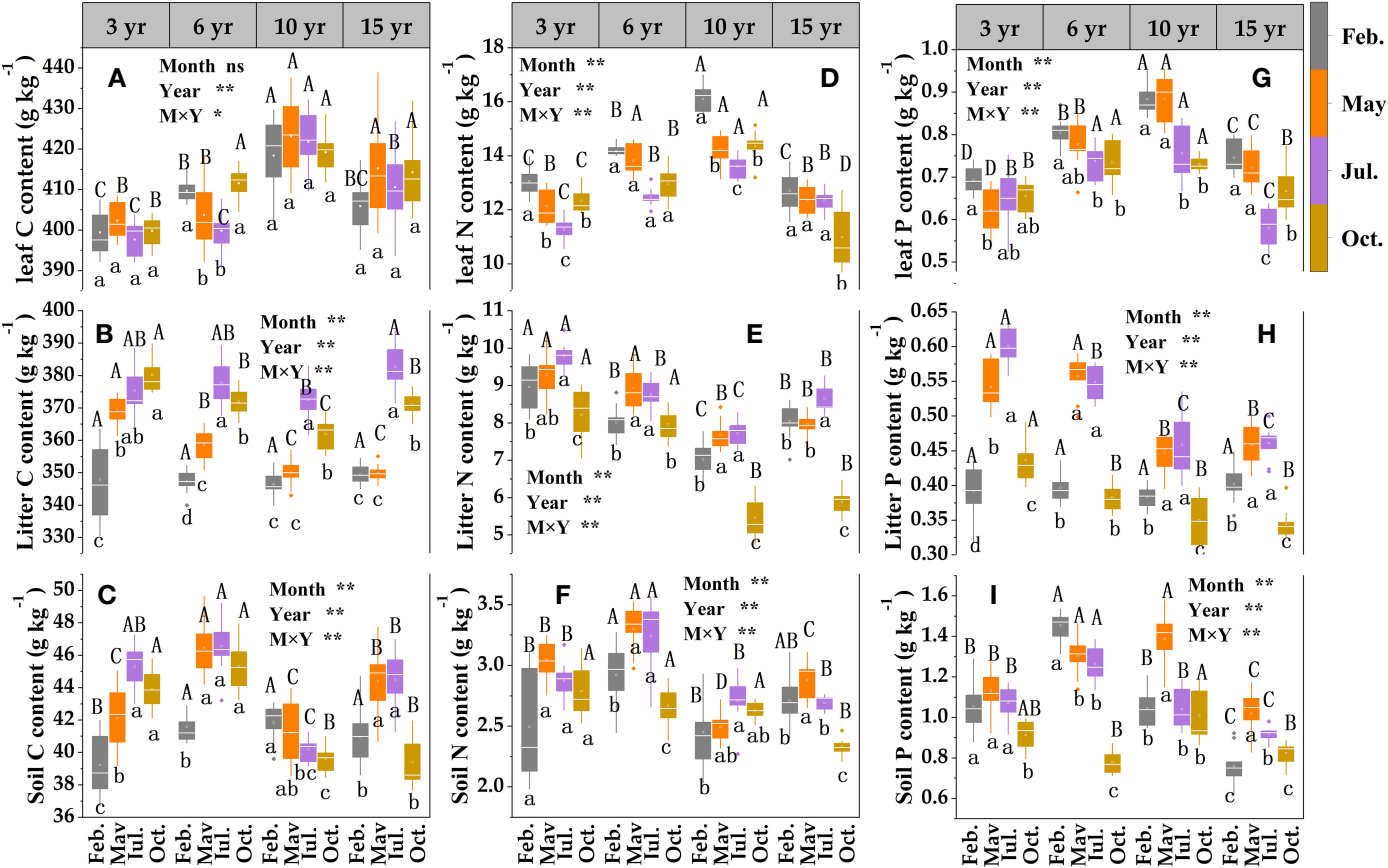
Content characteristics of leaf -litter- soil C **(A–C)**, N **(D–F)** and P **(G–I)** in loquat forests (n = 9). Different capital letters indicate significant differences between different sampling times (*P<* 0.05), and different lowercase letters indicate significant differences between recovery years (*P<* 0.05). ^*^ *P<* 0.05; ^**^ *P<* 0.01; ns, not significant.

The two-way ANOVA indicated that sampling month, restoration year, and their interaction had significant effects on leaf N, P, litter, and soil C, N, P contents. There was a general trend for the contents of C, N, and P in loquat leaves, in which they increased and then decreased with increasing years of restoration (**Figure 4**), reaching a maximum level at 10 years of restoration. The leaf C content did not differ significantly (except for 6 years of recovery) between sampling times (*P* > 0.05) (**Figure 4A**); the loquat leaf N content revealed a gradual decrease (except for 15 years of recovery) (**Figure 4D**), and the maximum mean value was in February. The lowest value of phosphorus, which was only 0.59 g/kg, among all treatments was in July at 15 years of recovery (**Figure 4G**).

The contents of litter C (**Figure 4B**) and P (**Figure 4H**) showed a tendency to first increase and then decrease with the increase in sampling time (except for the gradual increase in the restoration of 3 years). The P content differed significantly (*P<* 0.05) among treatments except in May and July, and the maximum value was in May or July. The litter N content revealed a total trend of decreasing and then increasing with the increase in restoration years, and the same restoration years revealed a tendency to increase and then decrease with the sampling time (**Figure 4E**).

The contents of soil C (**Figure 4C**), N (**Figure 4F**) and P (**Figure 4I**) at different restoration periods showed an initial increase followed by a decrease trend with the increase of sampling time (apart from the restoration of soil carbon after 10 years and soil phosphorus after 6 years); at the same sampling time they began to decrease after 6 years of restoration, with decreases of 10.47%, 18.01% and 7.06%, respectively, at 10 years of restoration compared with 6 years.

### 3.2 Plant-litter-soil ecological stoichiometry characteristics

**Figure 5:**
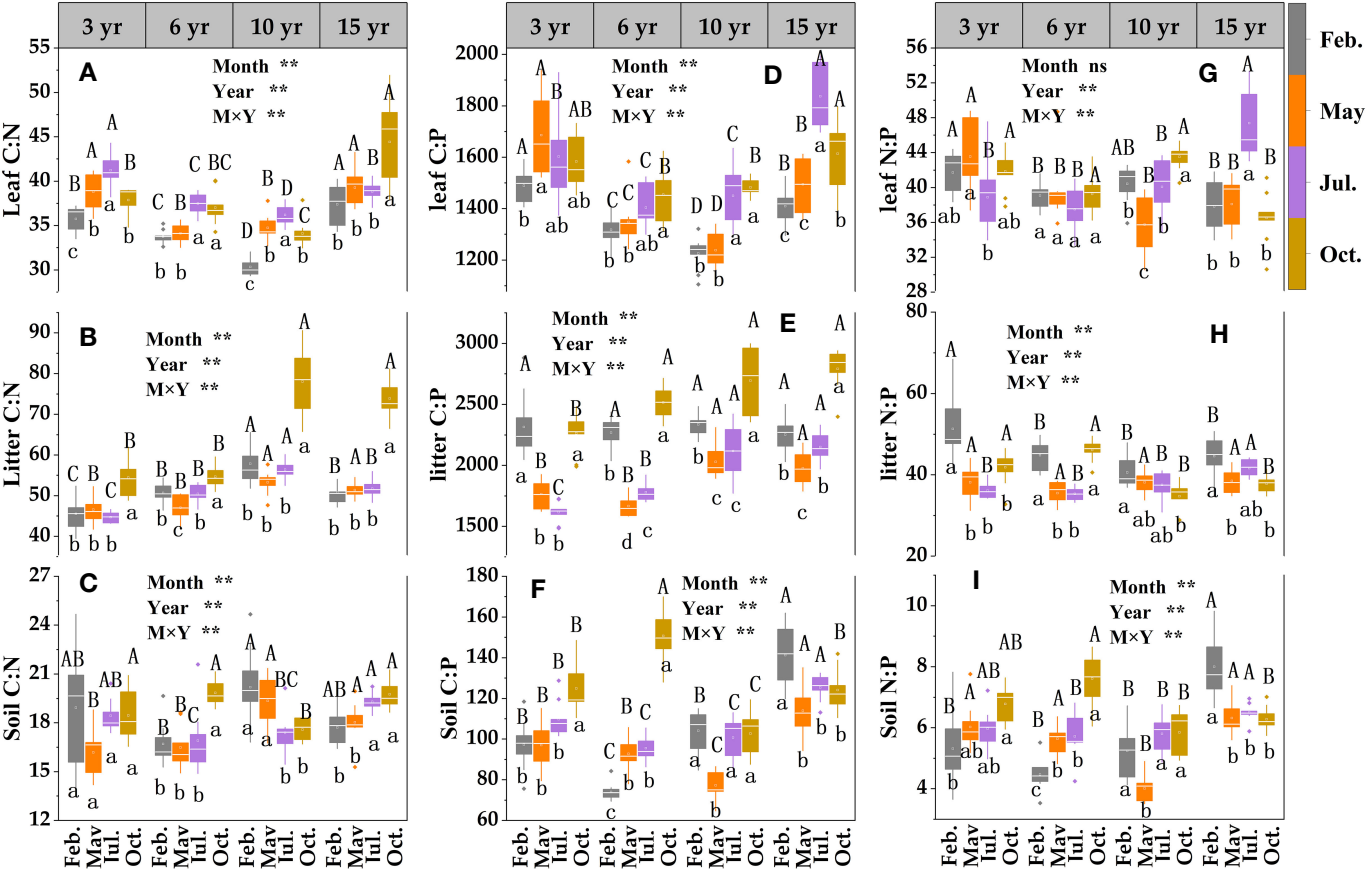
The stoichiometric ecological characteristics of leaf -litter- soil C:N **(A–C)**, C:P **(D–F)** and N:P **(G–I)** in loquat forests (n =9). Different capital letters indicate significant differences between different sampling times (*P<* 0.05), and different lowercase letters indicate significant differences between recovery years (*P<* 0.05). ^**^ *P<* 0.01; ns, not significant.

The two-way ANOVA indicated that sampling month, restoration year, and their interaction had significant effects on leaf C:N, C:P, litter, and soil C:N, C:P, N:P contents (**Figure 5**). The leaf (**Figure 5A**), litter (**Figure 5B**) and soil (**Figure 5C**) C:N values of loquat at different restoration years were as follows: litter > leaf > soil. The overall trend of leaf C:N decreased and then improved with the growth of restoration years and was the lowest at 10 years of restoration (**Figure 5A**). There was a growing trend and then a reduction with the increase in sampling time at 3, 6 and 10 years of restoration, and the maximum value was in July, which was significantly greater than that in February (*P<* 0.05). The litter C:N ratio showed a trend of decreasing and then increasing with increasing sampling time at 6 and 10 years of recovery, and the value in October was markedly (*P<* 0.05) greater than that in May (**Figure 5B**). The soil C:N decreased by 2.97% from 3 to 6 years of restoration (**Figure 5C**).

The C:P of leaf, litter and soil showed litter > leaf > soil in all restoration years (**Figure 5**). The leaf C:P revealed a decreasing tendency after increasing with increasing restoration years, and the values at 3 and 15 years of restoration were significantly higher than those at 6 and 10 years of restoration (*P<* 0.05) (**Figure 5D**). There was a gradual increasing trend with increasing sampling time at 6 and 10 years of restoration, and the maximum value in October was prominently greater than that in February. Both litter and soil C:P increased with increasing restoration years. The litter C:P ratio displayed a tendency towards decreasing and then increasing with ascending sampling time for the same restoration years and was observably greater in October than in May and July (**Figure 5E**). The soil C:P showed a gradual increase with increasing sampling time at 3 and 6 years of recovery, and the value in October was notably greater than that in the other months (*P<* 0.05) (**Figure 5F**).

The leaf, litter, and soil N:P ratios were 35.76-47.39, 34.73-51.29 and 4.01-8.00, respectively, at different restoration years, with an overall trend of litter > leaf > soil (**Figure 5**). The highest leaf N:P was in July of the 15th year of restoration, with a value of 47.39 (**Figure 5G**), which was prominently higher than that of the other restoration years in the same month (*P<* 0.05). The litter N:P had a trend of first decreasing and then with sampling time at 3 and 6 years of restoration (**Figure 5H**), and in February, it was significantly greater than that in May and July (*P<* 0.05). The N:P ratios of soil increased by 29.63% from 10 to 15 years of restoration (**Figure 5I**). There was a trend of a gradual increase with the increase in sampling time at 3 and 6 years of restoration, and the value in October was prominently greater than the value in February (*P<* 0.05).

### 3.3 Homeostasis analysis of P, N and N:P in loquat leaves with soil nutrient changes

**Figure 6:**
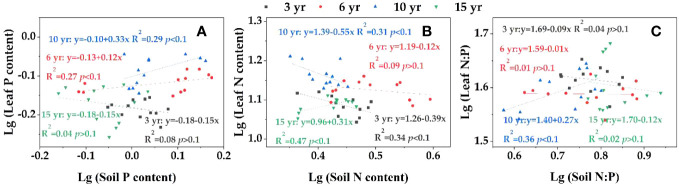
Homeostasis analysis of leaf P **(A)**, N **(B)** and N:P **(C)**.

The homeostasis characteristics were different in leaf N, P and N:P with soil nutrient changes during loquat recovery (**Figure 6**), among which the leaf P (**Figure 6A**) at 3 and 15 years of recovery, leaf N (**Figure 6B**) at 6 years of recovery, and N:P (**Figure 6C**) at 3, 6 and 15 years of recovery were not significant (*P >*0.1) and belonged to absolute homeostasis. The leaf P at 6 years of recovery belonged to the homeostasis index; the P at 10 years of recovery, the N at 3 and 15 years of recovery, and the N:P at 10 years of recovery had weak homeostasis with 1/*H* of 0.33, 0.39, 0.31, and 0.27, respectively. The N at 10 years of recovery was a weakly sensitive indicator with a 1/*H* of 0.55.

### 3.4 Relationships between leaf, litter and soil C, N, and P contents and C:N:P stoichiometry

**Figure 7:**
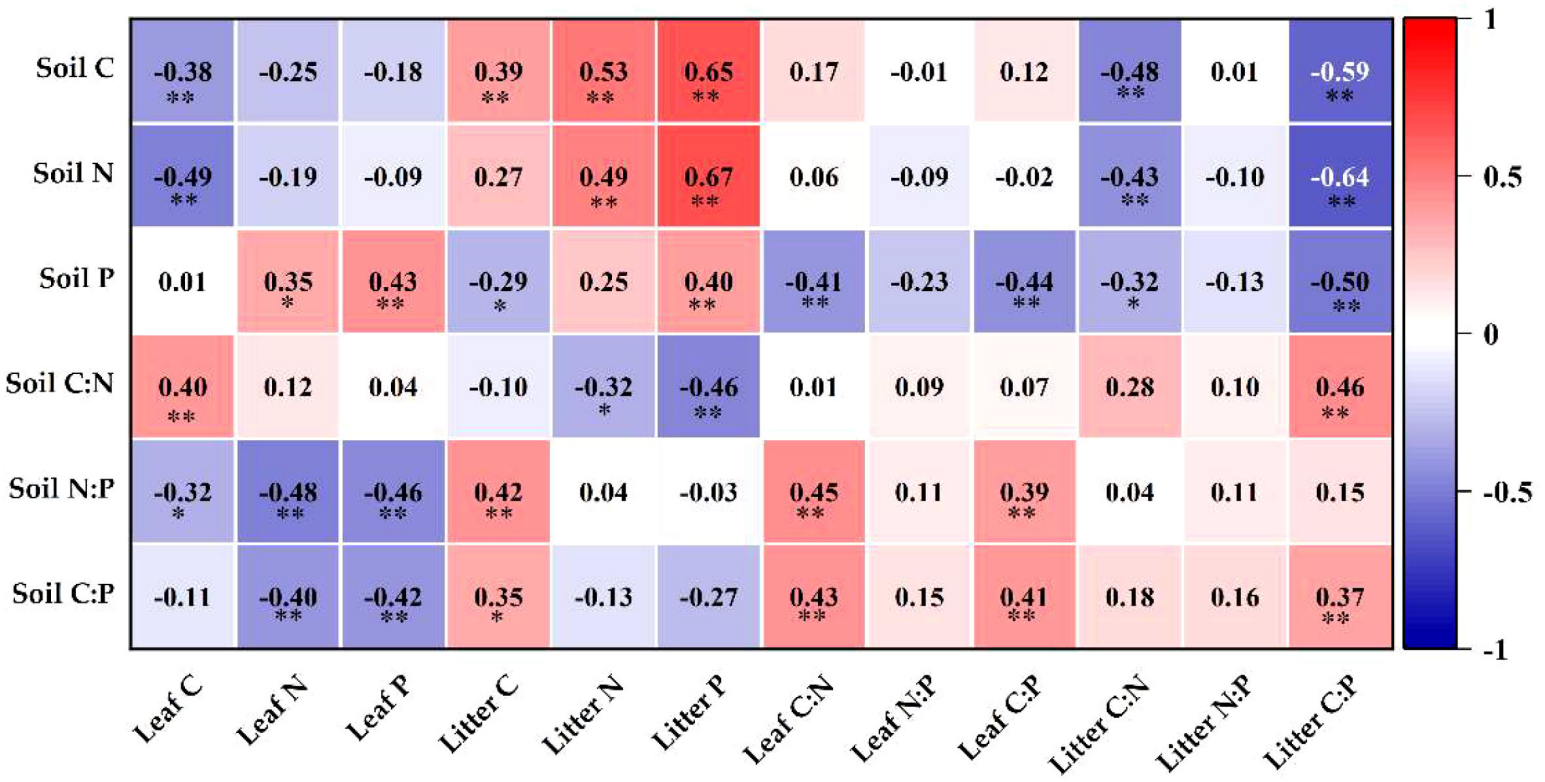
Graph of correlation analysis. **Correlation is significant at the level of 0.01; *correlation is significant at the level of 0.05.

The correlations between litter, leaf and soil nutrient contents and C:N:P stoichiometry are shown in **Figure 7**, where the following pairs were extremely significantly positively correlated (*P*< 0.01): soil C and litter C and N; soil N and litter N and P; soil C:N and leaf C; soil P and leaf and litter P; soil C:P and leaf and litter C:P; soil N:P and leaf C:N and C:P. Moreover, soil P and litter C:P, soil C and leaf C, and soil C:P and leaf P were highly significantly negatively correlated (*P*< 0.01). There were notable inverse relationships between soil N:P and leaf C, soil C:N and litter N, and soil P and litter C:P (*P*< 0.05), and soil C:P and litter C and soil P and leaf N were significantly positively correlated (*P*< 0.05).

## 4 Discussion

### 4.1 Vegetation restoration alters the C, N, and P contents of leaf-litter- soil in the control of karst desertification

C, N, and P are critical components of crop development and growth, and their coupling and cycling processes drive normal crop growth and sustainable ecosystem development (Hessen and Elser, 2005). It has been shown that the nutrient content among different forest ages shows a trend of middle-aged forest > mature forest > young forest (Ma et al., 2017). At the same sampling time, the C, N, and P contents of loquat leaves in different restoration years indicated a growth trend followed by a reduction with increasing restoration years in the study (**Figures 4A–C**). These results were consistent with other studies (Deng et al., 2019b), and the highest contents were found at 10 years, indicating that the physiological characteristics of loquat in the karst desertification control area reached their highest at approximately 10 years under a natural growth state. This may be related to the soil characteristics in the area, therefore, it is necessary to strengthen soil nutrient management. Litter is the hub linking vegetation and soil and is the primary means by which plants return nutrients to the ecological system (Kang et al., 2010; Yang et al., 2014). The study showed that the litter C, N, and P contents were different at different sampling times in the same restoration year (**Figure 4**), and the maximum value of the P content was in May or July, this may be associated with high rainfall and elevated temperatures in May and July. Indicating that litter may have different decomposition rates with temperature and humidity in different months, resulting in different nutrient stocks. Vegetation restoration will increase organic matter return and change soil nutrients through litter material (Liu et al., 2018). In this study, the maximum values of the soil N (**Figure 4H**) and P (**Figure 4I**) contents occurred at 6 years, indicating that the nutrients consumed by the plants increased as they grew over time. Soil nutrient management should be strengthened in advance in the vegetation restoration process for karst desertification control to prevent early plant decline.

### 4.2 Vegetation restoration changes the C:N:P stoichiometry characteristics in loquat forest ecosystems

Ecological stoichiometry is often used to search for the interrelationships between the plant and belowground parts of terrestrial ecosystems (Sophie et al., 2015; Damien et al., 2016). Leaf C:N:P stoichiometry characteristics can embody the ability of the plant body to obtain nutrients from the soil and utilize them. In this study, the C:N and C:P values of loquat leaves in the four recovery years were lower in February (**Figure 5**). The results indicate that loquat N and P use efficiency is higher in February, which may be related to less rainfall in February and less leaching of N and P. The growth velocity of biology shows a negative correlation with C:P and C:N according to the growth velocity hypothesis (Sterner and Elser, 2002). These results indicate that the leaf C:P and C:N tended to decrease after then increase with increasing restoration years, suggesting that the loquat growth rate tended to improve and then decline with increasing restoration years. Numerous studies have shown that the leaf N:P ratio may be applied to reflect whether the crop is P-limited or N-limited in an ecosystem (Richardson et al., 2008; Fan et al., 2015; Güsewell, 2004). In the present study, the leaf N:P ranged from 35.76 to 47.39 at different restoration years, which was greater than the national mean N:P (32) of plant leaves (Klausmeier et al., 2004). Available studies suggest that plant leaves N:P > 35 may be P-limited (Tessier and Raynal, 2003). Therefore, P limitation may exist during the growth of loquat in the study area. Scientists have found P limitation in karst areas such as Yunnan and Guangxi (Chen et al., 2018; Jiang et al., 2022). In this study, although the content of total P in the soil was not low, P limitation existed due to the alkaline nature of the soil in the karstic desertification management area and the high calcium carbonate content (**Table 2**), which makes P highly susceptible to soil fixation, resulting in low activity and low effective P content that can be taken up and used by crops.

**Table 2:** Soil pH value and its available nutrient characteristics (mean ± S.D., n=9).

Recovery year	pH	Alkaline nitrogen (mg·kg^-1^)	Available phosphorus (mg·kg-1)
3 yr	7.55 ± 0.24	113.78 ± 10.70	5.80 ± 0.74
6 yr	7.54 ± 0.10	122.83 ± 4.58	4.95 ± 1.29
10 yr	7.43 ± 0.18	131.35 ± 4.14	4.08 ± 1.54
15 yr	7.52 ± 0.23	143.86 ± 9.07	3.31 ± 0.82

The decomposition rate of litters was negatively correlated with the C:N (Sariyildiz and Anderson, 2003; Parton et al., 2007). In this research, the litter C:N increased significantly (*P*< 0.05) from 3 to 10 years of restoration (**Figure 5D**), showing that the litter decomposition velocity was higher in the early period than in the middle period of vegetation restoration. The C:N of litter in the research ranged from 44.75-78.03, between low C:N (12-20) and high C:N (108), and decomposers were less restricted by N (Vitousek, 1982).

Soil C, N, and P stoichiometry can effectively predict nutrient saturation status and play a significant role in ecosystem material cycling, elemental balance, and biological survival (Vitousek et al., 2010; Du and Gao, 2021). The soil C:N ratio ranged from 16.49-20.19, which was greater than the global mean (13.33) and the Chinese mean (11.0) (McGroddy et al., 2004; Tian et al., 2010). The soil C:P (**Figure 5F**) and N:P (**Figure 5I**) ratios showed gradual increases with increasing sampling time at 3 and 6 years of recovery, and the values in October were notably greater than those in February (*P<* 0.05). This difference may be due to increased litter decomposition leading to C and N inputs to the soil.

### 4.3 Relationship between loquat leaves, litter and soil C:N:P traits and homeostasis

Soil is an important basic condition for the growth of plants, and the nutrient content and ecological stoichiometry are influenced by soil nutrients (Hu et al., 2017; Li et al., 2022). An analysis of soil, litter and leaf C, N and P contents and their stoichiometric relationships revealed that soil N and litter N and P, soil C and litter C and N, soil P and litter and leaf P, soil C:N and litter C:P, soil C:P and leaf and litter C:P, and soil N:P and leaf C:P and C:N had highly significant positive relations (*P*< 0.01) (**Figure 7**), indicating a close coupling between soil nutrient contents and ecological stoichiometry characteristics in vegetation restoration in karst desertification management. When soil nutrients are altered, plants maintain the stability of their internal chemical elements through homeostasis regulation (Sterner and Elser, 2002; Yu et al., 2011). Except for the recovery of N at 10 years in this study, which was weakly sensitive, the others belonged to absolute homeostasis, homeostasis and weak homeostasis (**Figure 5**), indicating that loquat is highly adaptable and can well regulate its internal physiological metabolism to adapt to the arid and barren environment in karst desert areas.

## 5 Conclusions

Loquat leaf C, N and P contents were the highest in year 10. The decomposition rate of litter in early vegetation restoration was higher than that in the medium period, and the rate of decomposition of litter varied with temperature and humidity at different sampling times, which led to different nutrient reserves. The maximum C, N, and P contents in soil for the same recovery years were mostly in May and July, possibly due to increased nutrient breakdown and transformation due to high water content in the environment. Loquat may face P restriction during the growth process, perhaps due to the low effective P content in soil. The relationship between leaf N, P and N:P and soil indexes can be simulated by a homeostasis model. Plants maintain homeostasis by regulating their internal physiological responses in the face of soil nutrient changes, indicating that loquat has good adaptability in karst desertification environments, but the balanced management of soil nutrients should be considered in the process of vegetation restoration. In particular, some agronomic measures should be taken to improve soil P availability. Therefore, to ensure that the results of karst desertification control can play a role in rural regeneration, future research should apply scientific management that takes into account soil moisture and nutrients in different seasons.

## Data availability statement

The original contributions presented in the study are included in the article/supplementary material. Further inquiries can be directed to the corresponding author.

## Author contributions

TH: writing original draft. KX: validation, formal analysis, and investigation. JW: writing review and editing. YW: visualization and editing. YY: editing. All authors contributed to the article and approved the submitted version.
